# Printed Twisted Thin Films with Near-Infrared Bandgaps and Tailored Chiroptical Properties

**DOI:** 10.1021/acsaom.4c00386

**Published:** 2024-11-21

**Authors:** Botyo Dimitrov, Daria Bukharina, Valeriia Poliukhova, Dhriti Nepal, Michael E. McConney, Timothy J. Bunning, Vladimir V. Tsukruk

**Affiliations:** † School of Materials Science and Engineering, Georgia Institute of Technology, Atlanta, Georgia 30332-0245, United States; ‡ Air Force Research Laboratory, Wright-Patterson Air Force Base, Dayton, Ohio 45433, United States

**Keywords:** cellulose nanocrystals, preprogrammed helicity, NIR reflectance, active circular polarization, discrete optics, dual circular dichroism, twisted dielectric mirror

## Abstract

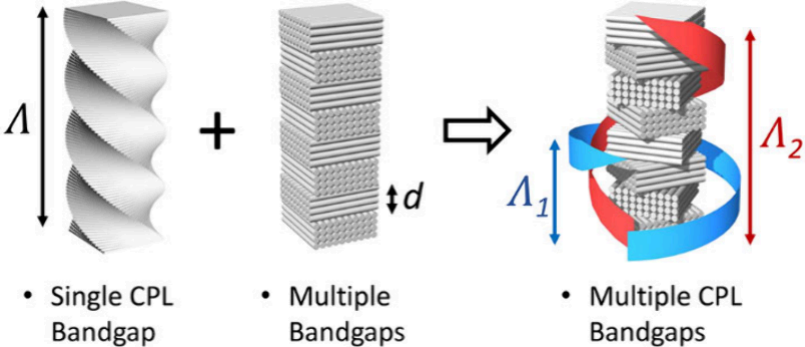

In this work, twisted helical cellulose nanocrystals films with preprogrammed circular polarization and near-infrared reflectance are fabricated via a blade-based 3D printing method. The films are composed of stacked nanoscale slabs with high birefringence from unidirectionally organized cellulose nanocrystals. By changing the printing director, we achieved two types of films: twisted helical stacks and anisotropic Bragg stacks. These films are highly transparent and clear, and the achiral anisotropic Bragg stack shows near-infrared spectral region reflectance (1.3–1.4 μm). In contrast, the twisted helical films show concurrent left- and right-handed circularly polarized properties, as opposed to left-handed natural cellulose nanocrystals films. We observe dual chiroptical properties with circular dichroism peaks due to circular Bragg reflectance in the visible region and suggest that the circularly polarized properties are extended to the near-infrared region. These observations prompted us to explore the transition between anisotropic Bragg stacks and continuous helical films via simulations. We show that the printed twisted films can act as optical metamaterials with dual helicity and fill the gap between known photonic structures—the conventional continuous chiral nematic material with a chiroptical appearance and the achiral Bragg stack with a controlled photonic bandgap. These printed twisted stacked films hold the potential of larger-scale printed ordering of unique anisotropic nanostructures for circularly polarized-sensitive photonic, thermal, and energy management applications.

## Introduction

More than 40% of the world’s energy consumption is spent on heating, cooling, and ventilation.^1^ Solar heat gain through windows accounts for 20–40% of energy losses in buildings and vehicles.^2^ Research on new smart window technologies, aiming at reducing solar irradiation, and decreasing the thermal footprint has significantly increased in the last 10 years,^3^ and the field has seen substantial commercialization via electrochromic windows, polymer dispersed liquid crystals (PDLC), and suspended particle device (SPD) technologies.^4^ However, current smart windows are based on absorption and scattering of incident light over a broad spectral range spanning the UV, visible, and near-infrared (NIR) regions. Although broadband light absorbance is an efficient approach for adjusting solar irradiation, it comes at a cost in terms of occupant comfort: visible light is also excluded, resulting in a loss of view, with a final result similar to that of conventional mechanical shaders.^5^

Nearly one-third of total spectral energy comes from the visible range, while two-thirds is spread over a wide NIR range.^6^ Therefore, to achieve energy and thermal efficiency without losing visibility, it is of interest to maintain a good transmittance of visible light and selectively control the transmittance of NIR radiation. Photonic crystal organizational principles are an appropriate approach to address the selectivity requirement because light–matter interactions depend on the submicron scale refractive organization of the material,^7−9^ rather than absorption and plasmonic phenomena due to electron excitation.^10,11^ Geometrical periodicity with proper refractive index distribution allows for precise control of the optical properties and therefore is a suitable candidate to selectively transmit or reflect specific wavelengths.^9,12^ With a circularly periodic organization, helical (chiral nematic, cholesteric) liquid crystals (LC) are an actively researched materials system capable of satisfying the targeted reflectance requirements due to their versatility, tunability through chiral dopants, and stabilization via cross-linking.^13,14^ Chiral nematic LC films exhibit a maximum of 50% reflectance at bandgap wavelengths, owing to the selective reflection of light with the same handedness, e.g., left-handed (LH) structures will reflect LH circularly polarized light (CPL). Thus, in an LH film, nonpolarized light will be decomposed to reflected LH and transmitted right-handed (RH) CPL. There has been an interest in overcoming the narrow bandgap limitations of chiral nematic LC materials, originating from the low in-plane refractive index contrast,^15^ by introducing a gradient pitch length. It has been demonstrated that inducing a concentration gradient of a chiral dopant could lead to a gradient pitch length throughout the film thickness.^16^ The graded material exhibited broad reflectance over the NIR and visible ranges.^17^

Alternative natural polymer-based photonic materials have been thoroughly investigated in recent years due to their renewable sourcing and sustainability potential.^18−22^ Colloidally dispersed cellulose nanocrystals (CNCs) in water can form a lyotropic liquid crystalline (LC) phase^23,24^ and can self-assemble via slow evaporation in ambient room conditions into a solid chiral nematic film.^25^ Attempts have been made to achieve responsive films through dynamic pitch length change driven by changing environmental conditions or mechanical stress.^26,27^ Other research has been focused on achieving NIR reflection via the intercalation of additives, resulting in CNC-based helical composites. For instance, organosilica additives were used to achieve a sufficient pitch shift and NIR reflectance.^28^ In a different set of studies, the common plasticizer polyethylene glycol was used to improve the mechanical robustness by serving as an intercalator^29^ for a sandwich structure of cholesteric–nematic–cholesteric film to achieve a reflector for both LH and RH CPL.^30−32^ Other bottom-up approaches have included self-assembly in a magnetic field^33,34^ and solvent exchange.^35^

However, to date, bottom-up approaches aiming at red-shifting cholesteric CNC films have yielded a lower reflectance due to the emergent disorder, thus perturbating the periodicity of the CNC arrangement. On the other hand, top-down approaches could present an alternative manufacturing route to maximize the regularity of the helical structure. Previous research on layered helical structures has used the Langmuir–Blodgett technique to orient nanowires unidirectionally to achieve structurally colored films with distinct pitch lengths and reflectance peaks.^36^ Recent results on shearing lyotropic LC CNC suspensions have demonstrated the assembly of a unidirectionally oriented CNC film,^37^ and the same shearing method has also been shown to achieve uniaxial nematic-like organization with CNC and other lyotropic suspensions.^38−41^

Twisted anisotropic helical media have been previously theoretically modeled and predicted to result in circular Bragg bandgaps.^42,43^ Recently, the appearance of multiple reflectance peaks has been explained. Hu et al. have shown via simulations that helically organized anisotropic layers will yield both LH and RH reflectance peaks. By varying the twisting angle, the location of the circular Bragg bandgaps can be controlled.^44^ In our previous work, we investigated the layering of CNCs via blade coating to engineer the helicity of the films, and we demonstrated that both LH and RH circular dichroism (CD) are possible depending on the helicity of the layered film. The utilized blade coating technique achieved highly oriented CNC films with controllable layer thickness.^45^

**Figure 1:**
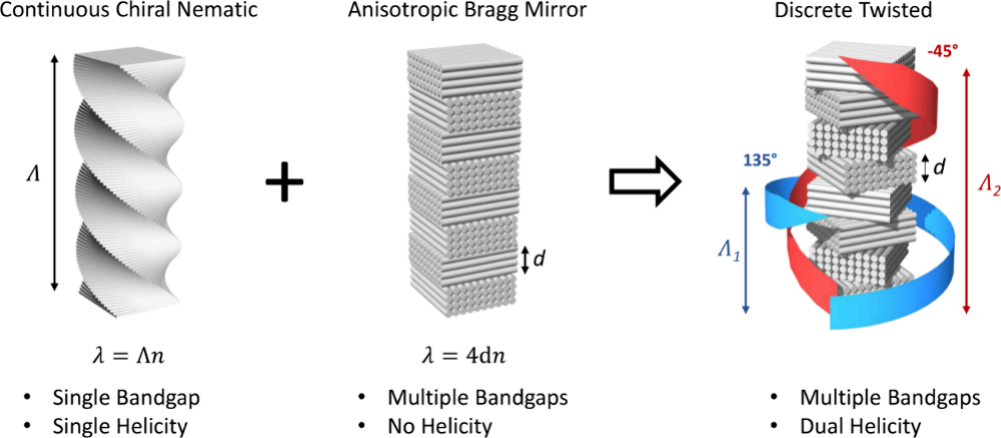
Discrete twisted materials: the combination of chiral nematic organization and anisotropic Bragg mirror stack results in dual optical chirality due to bidirectional rotational periodicity.

Using these results as a foundation, the present work investigates organized CNC films composed of shear-deposited anisotropic nanoscale blocks with periodically varying orientation to achieve a tailored helical CNC organization with preprogrammed pitch length and twisting angle (Figure 1). In contrast to self-assembled films, which have a chiral nematic gradual azimuthal orientation change of the CNCs, the produced preprogrammed printed films are discretely layered, with a finite layer thickness on the hundred nanometers scale and discrete angular difference (twisting angle) between sequential layers on the order of tens of degrees. Building on the previously suggested printed layering technique in our group,^45^ we experimentally demonstrate nontraditional dual periodic CD properties, which can be rationalized with simulations of discrete blocks with periodic optical constants.

Via control of the printing parameters, different element thicknesses and twisting angles can be accomplished, presenting a route to accurate top-down control of the final pitch length and, thus, the reflected spectral ranges and circular polarization properties. The dual CD properties demonstrated here open avenues for top-down structuring based on two controllable macroscale parameters, substrate rotation and suspension volume, to tailor chirality-sensitive optics in an accessible manner. Furthermore, the same 3D printing shear-deposition method is used to produce a material with NIR reflectance, essentially an anisotropic Bragg mirror, yielding novel NIR-reflecting materials.

It is important to note the distinction between the origins of the circular dichroism of chiral molecules and that from the discrete helical stacks of twisted blocks demonstrated here. In biological and organic molecules, molecular chirality is critical in distinguishing chiral isomers of small molecules,^46^ proteins,^47^ and supramolecular structures,^48^ and it occurs in chiral center-containing molecules due to the lack of mirror symmetry.^49^ On the other hand, CD was demonstrated on a large scale in artificially structured materials with a direction-dependent response^50−52^ and stacked twisted structures.^53,54^ Also, various molecules assemble in twisted helicoidal ribbons, and research has focused on their characterization by using Mueller matrix polarimetry.^55,56^ Metamaterial stacks and Moiré patterns can be formed from linear or 2D patterns,^57,58^ localized stacks from individual particles,^59^ and achiral layers such as graphene.^60^ In that sense, the twisted superstructures studied in this work, and in particular discrete twisted helical films, fulfill the condition of 3D helical organization but represent a distinct class of discrete optically active media.

## Experimental Section

### Cellulose Nanocrystals

CNCs were obtained via sulfuric acid hydrolysis from pressed wood pulp, yielding suspensions with concentrations varying between 0.5 and 1.5 wt % according to the established routine.^61^ The morphologies of CNCs and their assemblies were characterized thoroughly with the usual means.^62^ The CNC suspensions were then saturated by evaporation at an elevated temperature of 40 °C until a concentration of 5 wt % is obtained. Individual nanocrystals had an average length of 135 nm and a diameter of 4 nm (see Table S1).

### Layered Shearing Printing with Blade Coating

**Scheme 1:**
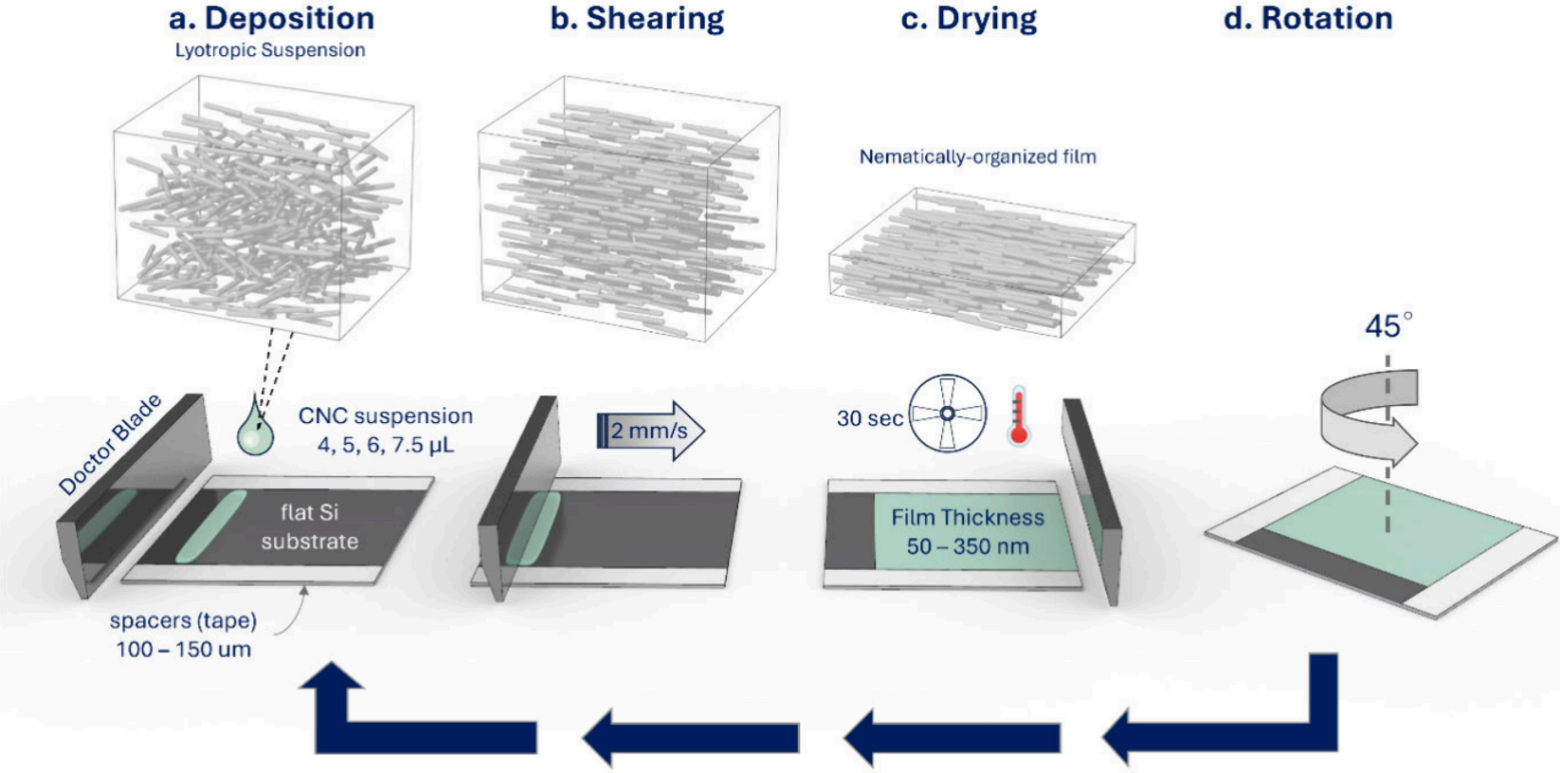
Printing of Discrete Twisted Films by Layer-by-Layer Rotation of Shearing Direction: (a) Deposition of the Suspension, (b) Shearing of the Suspension with a Blade Coater, (c) Drying with Hot Air, and (d) Rotation of the Substrate with the Dried Layer Steps (a)–(d) can be repeated to achieve a layered film.

A TQC Sheen blade coater was used for printing multilayered twisted films as introduced recently (Scheme 1 and Figure S1).^45^ The substrate was attached to the blade coater stage with tape, which also served as a spacer between the substrate and the blade. This method is based on deposition of the material suspension followed by immediate drying, which causes water evaporation and a significant decrease of the layer thickness. The thickness of a single tape was ∼50 μm, which is orders of magnitude greater than the final film thickness of 50–350 nm (Figure S3). A shearing rate of 2 mm/s was used for all samples.

As discussed in our previous study, the 5 wt % concentration of the CNC suspension yielded the most uniformly oriented layers. While other CNC concentrations (from 0.7 to 8.6 wt %) also resulted in flat dry layers, they lacked a high orientational order parameter.^45^ Therefore, in this work, we investigated the stacking of layers produced by depositing 5 wt % CNC suspensions. In addition to the CNC layer, a 0.5 wt % polyethylenimine (PEI) solution was used for the formation of a bilayer, with the PEI deposited before each CNC layer.

The positively charged PEI acts as a stabilizer, improving the quality of the next CNC layer, and does not contribute to the optical polarization.^63^ Fused silica and atomically flat silicon were used as the substrates. It is worth noting that the CNC suspension is shear thinning due to the 1D shape of the CNCs, and the uniformity and thickness of the final film depend on the shearing rate. After demobilizing the sample on the surface, applying the spacers, and carefully positioning the blade, the suspension was deposited at the blade location using a pipette. The blade coater was then started, continuously shearing the suspension along the substrate. After deposition, drying with a heat gun was done for up to 30 s in order to preserve the CNC orientation.

Single bilayer films were produced with 5 μL of PEI solution and 4, 5, 6, 7.5, and 2 × 4 μL of CNC suspensions (designated as CNC4, CNC5, CNC6, CNC7.5, and CNC8). The multilayered twisted helical films containing a preprogrammed number of PEI-CNC bilayers were fabricated as follows. After deposition of the first bilayer, the substrate was rotated at 45° (or 90°). Then, the next bilayer was deposited. The rotations were repeated with the same angular step, so the relative angle between the two consecutive layers was kept the same across the whole film. The final result after multiple depositions was a twisted structure of uniaxially anisotropic CNC layers separated by the PEI layer. Samples with 5 μL of PEI solution and 4, 5, 6, 7.5, and 2 × 4 μL CNC suspension (2 sequential depositions of 4 μL each) with LH helicity were produced (designated as CNC4-45, CNC5-45, CNC6-45, CNC7.5-45, and CNC8-45). All helical samples were made with 8 steps (twists) to form a single pitch length. Additionally, films with 90° twisting were prepared with 10, 14, and 22 layers with 7.5 μL of suspension (designated as CNC7.5-90).

### Atomic Force Microscopy (AFM)

Surface morphology and film thickness were investigated with a Bruker Dimension Icon atomic force microscope. Measurements are done in “Standard Tapping” mode in air with standard AFM tips, with 512 samples per line and a scanning rate of 0.5 and 1 Hz, on 5 × 5 μm and 20 × 20 μm regions according to the usual routine.^64^ Postprocessing for image flattening, depth measurements, cross-sectional height, and microroughness calculations was performed using the NanoScope Analysis 2.0 software.

### Scanning Electron Microscopy (SEM)

Cross-sectional images of the films were obtained using an 8230 FE-SEM with a cold field emission gun in secondary electron detection mode at a working distance of 5–10 mm and 3 kV voltage. To obtain the cross section, the sample was cut with a diamond cutter and then broken into two pieces. After that, the sample was coated with Au:Pd using a Q150 V Plus automatic coater and attached with carbon tape to a 90° stub.

### UV–Vis–NIR Spectrophotometry

Transmittance spectra of the films were obtained with a Shimadzu UV-3600 Plus instrument. The fused silica substrate samples were measured with a single beam measurement without a reference sample in the range 300–1600 nm with a step of 1 nm.

### Spectroscopic Ellipsometry

For determining the anisotropic refractive index of the CNC film, ellipsometry measurements were performed with a J.A. Woollam Co. M-2000U spectroscopic ellipsometer. The single-layer CNC film was simulated as a Cauchy dielectric with no extinction coefficient and with biaxial anisotropy. Model parameter fitting was performed with the WVASE software.

### Optical Thickness Estimation

As a complementary method to the AFM scratch test, the film thickness was estimated by the Swanepoel method and fast Fourier transform (FFT) analysis, assuming the predominant interference will result from the top and bottom film interfaces, and by using the results UV–Vis–NIR data (Figures S10 and S11).

### Circular Dichroism Spectroscopy

CD was measured using a Jasco J-815 spectropolarimeter for all samples on a fused silica substrate in the range 200–1000 nm with a step of 1 nm.

### Photonic Simulations

Finite-difference time-domain (FDTD) simulations were performed for transmittance and CD properties in the ANSYS Lumerical (version 2023 R2.1) 3D FDTD solver software environment. Each layer was represented as a uniform diagonally anisotropic material based on the physical uniaxial optical anisotropy. Periodic lateral boundary conditions were applied by assuming an infinite film. The transmittance of unpolarized light was obtained indirectly via a “Field and Power Monitor” and by averaging the signal from transmitted S and P polarized light sources. A CPL source was simulated using the linear combination of S and P sources with a + or −90° phase shift. Finally, CD spectra were calculated by using the transmittance data from RH CPL and LH CPL incident light. In the simulated spectra, peak broadening occurred due to the finite mesh cell size and the resulting imperfection at the interfaces between layers.

A single-pitch-length film was simulated by the sequential layering of slabs with the same 140, 170, and 190 nm thickness as derived from experimental measurements, with each slab having a rotated permittivity attribute—a standard procedure within the software environment. These virtual films were used to simulate the CD of the twisted helical films. Multistacks containing sequentially stacked single-pitch-length twisted helical films were modeled by duplication and translation with a step of a single pitch length. The total thickness of the multistacks used for the transmittance simulations was equal to 20 pitch lengths. The anisotropic Bragg stack was simulated with a slab thickness of 220 nm.

Finally, a simulation of CD spectra was also performed on twisted models with increasing twisting angles and thickness steps with a fixed pitch length. For that purpose, a single pitch length (Λ = 1520 nm) and the corresponding 360° twisting were divided into 100, 50, 20, and 8 layers. CD spectra were obtained using the procedure described above. The effects of changing the twisting angle (5°, 15°, 30°, 45°, and 60°) while keeping a constant slab thickness (*d* = 190 nm) and total film thickness (1520 nm) were also investigated.

## Results and Discussion

### Single Bilayer Printing and Twisted Helical Films

**Figure 2:**
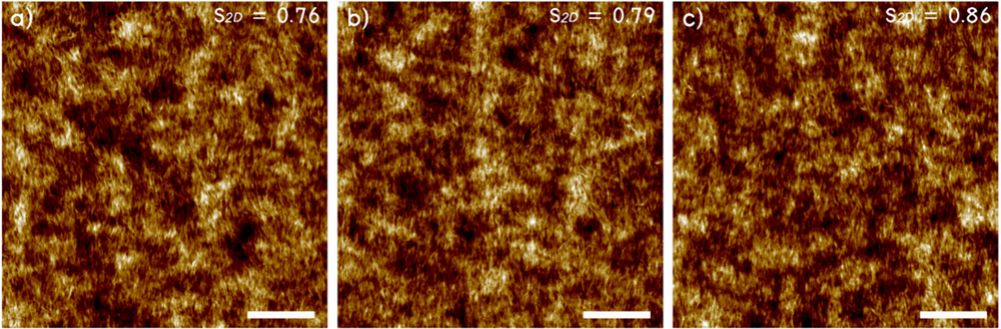
(a, b, c) AFM topographical images of CNC4, CNC5, and CNC6 single bilayer films. CNCs are approximately oriented in the direction of shearing, pointing north. The 2D orientational order parameter is shown in the top right corner. Scale bars are 1 μm.

First, we investigated the CNC4, CNC5, CNC6, CNC7.5, and CNC8 single bilayer films, which were used for deposition and fabrication of the twisted films in a layer-by-layer manner, as discussed in detail in our prior study.^45^ Our shear-printed layers were mechanically stable and showed highly unidirectional morphology of the film surface, with a high 2D order parameter *S*_2D_ within 0.76–0.86, as derived from orientational image analysis (Figure 2, Figures S2 and S6).^45,65^ These observations agree with our past research, in which we investigated the properties of lyotropic CNC suspensions and their properties after confined drying.^66,67^ The induced unidirectional orientation was also observed in other studies of sheared CNC suspensions.^37,39,40^ As expected, the produced bilayers have a growing layer thickness with increasing suspension volume, which was verified using an AFM scratch test (Figure S3). All printed films show very low microroughness (RMS values within 2 to 3 nm as measured on a 1 × 1 μm surface area),^64^ which is a condition for low scattering and high-quality optical properties (Figure S7).

Because of the highly oriented CNCs, the layer can be approximated to birefringent homogeneous media for wavelengths much larger than the nanocrystals. For the uniaxially anisotropic CNC film, the ordinary direction with refractive index *n*_o_ corresponds to the direction perpendicular to the CNC long axis. The extraordinary direction with refractive index *n*_e_ is parallel to the shearing direction and parallel to the CNC long axis. Ellipsometry data analysis shows a refractive contrast (Δ*n*) of 0.07 between *n*_e_ and *n*_o_ (Figures S4 and S5). The results are consistent with previous research on uniformly oriented CNC films.^68^

**Figure 3:**
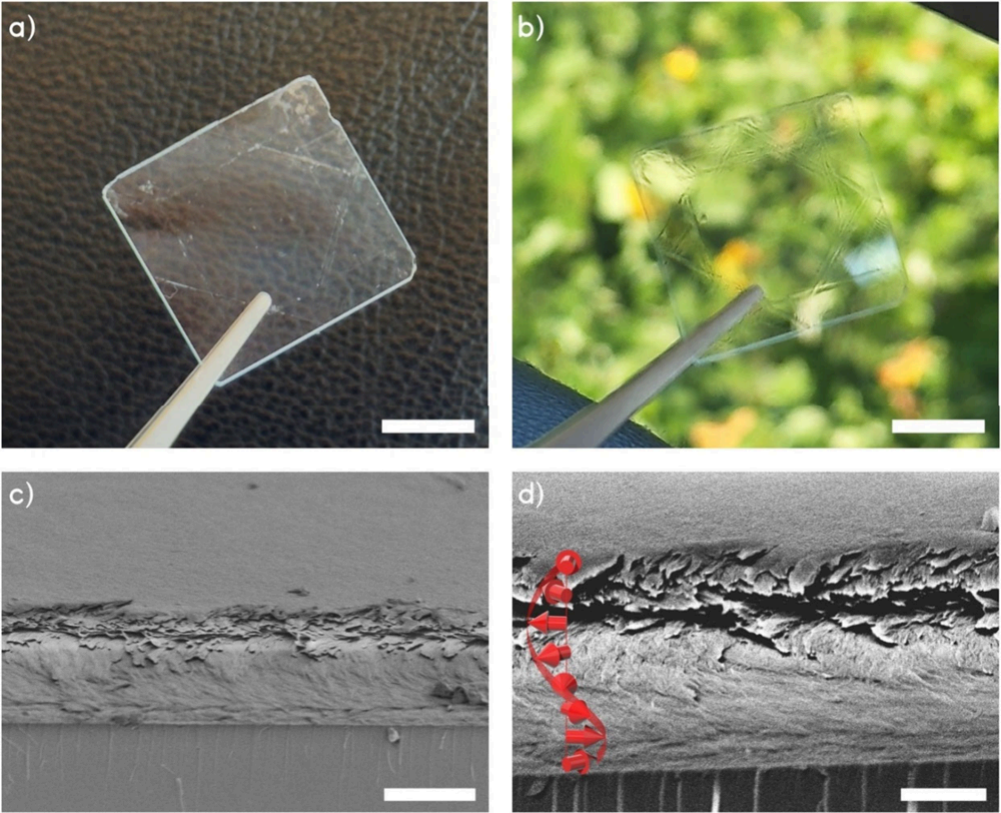
(a, b) Photographs of the CNC6-45 film on a fused silica substrate. (a) Film against a patterned background and (b) against outdoor ambient light. Material build-up can be seen on the edges of the film as diagonal and parallel lines. (c, d) Cross-sectional SEM images of the CNC6-45 film. The upper layers of the film are partially delaminated. The red helical arrows in (d) show the orientation of CNCs in the layers. The scale bars for (a, b) are 1 cm, and the scale bars for (c) and (d) are 2 and 1 μm, respectively.

Using the blade coating procedure with a rotating printing direction, we consistently produced twisted helical films with different bilayer thicknesses and preprogrammed 45° and 90° twisting angles (Scheme 1). These final films have a high transparency of approximately 91% in the visible range (Figure S9). The thickness of the CNC4-45 and CNC7.5-45 samples was measured via an AFM scratch test, showing 1.5 and 1.75 μm, respectively (Figure S8). Optical methods were also employed for the thickness estimation (Figures S10 and S11, eqs S1 and S2). The films are colorless, remarkably clear, and nearly indistinguishable from the transparent glass substrate (Figure 3a,b). These optical properties were preserved for extended periods.

For confirmation of the films’ internal structure, we conducted cross-sectional SEM studies (Figure 3c,d). The CNC films have a noticeable helical orientation, which varies through the height of the cross section in a regular pattern: from parallel to the section plane in the lower layers to perpendicular in the middle layers and so on, continuing to the top surface in a helical fashion, which is the so-called Bouligand morphology. There are no visible gaps between the layers, and the director vector that indicates the orientation is periodically rotated as shown in Figure 3d. This LH helical twisting organization is in close accordance with the design of the film and the rotational deposition sequence.

### Chiroptical Properties of Twisted Films

**Figure 4:**
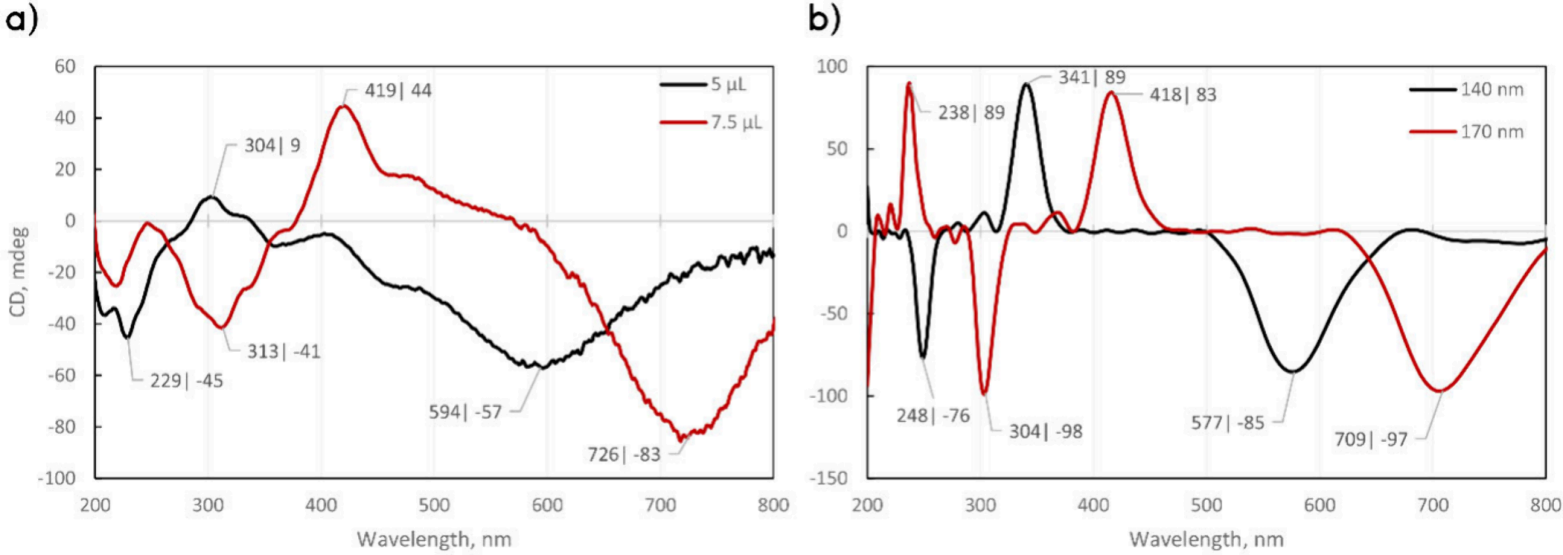
(a) CD experimental results for CNC5-45 and CNC7.5-45 and (b) FDTD simulations for 140 and 170 nm layer thicknesses, shown with black and red curves, respectively. Multiple positive and negative peaks and red-shifts with increasing layer thickness are detected.

Transmittance CD measurements show the presence of several positive and negative peaks in the UV and visible ranges (Figure 4a). A similar result was also obtained with FDTD simulations (Figure 4b). The peaks red-shift with increasing deposition volumes. For example, in Figure 4a, the peak at 594 nm for CNC5-45 is shifted to 726 nm for CNC7.5-45. The simulations in Figure 4b also show red-shifts for other peaks (e.g., from 577 to 709 nm) with growing layer thickness. It has to be noted that different CNC batches could result in varying layer thicknesses and can lead to slightly variable CD peak patterns (Figure S13), which are also highly sensitive to the shear-printing procedure. However, the presence of distinct positive and negative peaks points to the chiral organization of the films. The CD can be attributed to the helical twisting geometry, which is the reason for the emergence of chirality in the overall film, although the individual layers have a 2-fold mirror symmetry in the 2D plane.

It is important to note the significant difference in the optical phenomena discussed here compared to those of CD arising from chiral molecules. As it is well-known, molecular chirality CD, which in its nature is an induced redistribution of the electronic configuration in chiral molecules, causes resonances and absorption at specific wavelengths, which result in circular polarization.^49^ In contrast, the twisted helical dielectric photonic crystal considered here is a reflective mirror forming bandgaps for specific polarizations and wavelengths due to periodically alternating layers with optical anisotropy, described by photonic bandgap theory.^43^ In that sense, the peaks can be assigned to LH and RH twisting (helicity), rather than to the presence of a chiral phase (in the molecular sense) within the layers.^44^ The increase in CPL absorbance/reflectance, which gives rise to the CD peaks, occurs due to the rotationally induced periodicity via helical-like twisting. Because of the anisotropic slab organization, the film also acts as a multistack linear retarder. Therefore, we can also expect contributions from linear birefringence (LB) and linear dichroism (LD). LB and LD could yield partial conversion of the CPL to a different polarization state and are considered to be the reason for common artifacts. In our previous work, Mueller matrix spectroscopy was used to decouple the CD, LB, and LD.^45^

Another important consideration is that the measurements in the present work are focused on the overall differential transmittance of LH and RH CPL for a CD defined here as
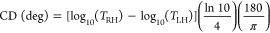
1where *T*_RH_ and *T*_LH_ are the transmittances of initial RH and LH CPL, respectively, regardless of the polarization state after passing through the film. The 45° twisting step in the LH film is expected to result in a positive LH CD peak. However, in the measured films, distinct peaks of both handedness occur in contrast to continuous chiral nematic media and self-assembled CNC films, which exhibit a single CD peak, with handedness corresponding to their helicity. To explore the role of the twisting angle magnitude and the nature of the observed peaks, a series of FDTD simulations with varying twisting angles were further performed.

### Transition from Continuous to Discrete Helical Films

**Figure 5:**
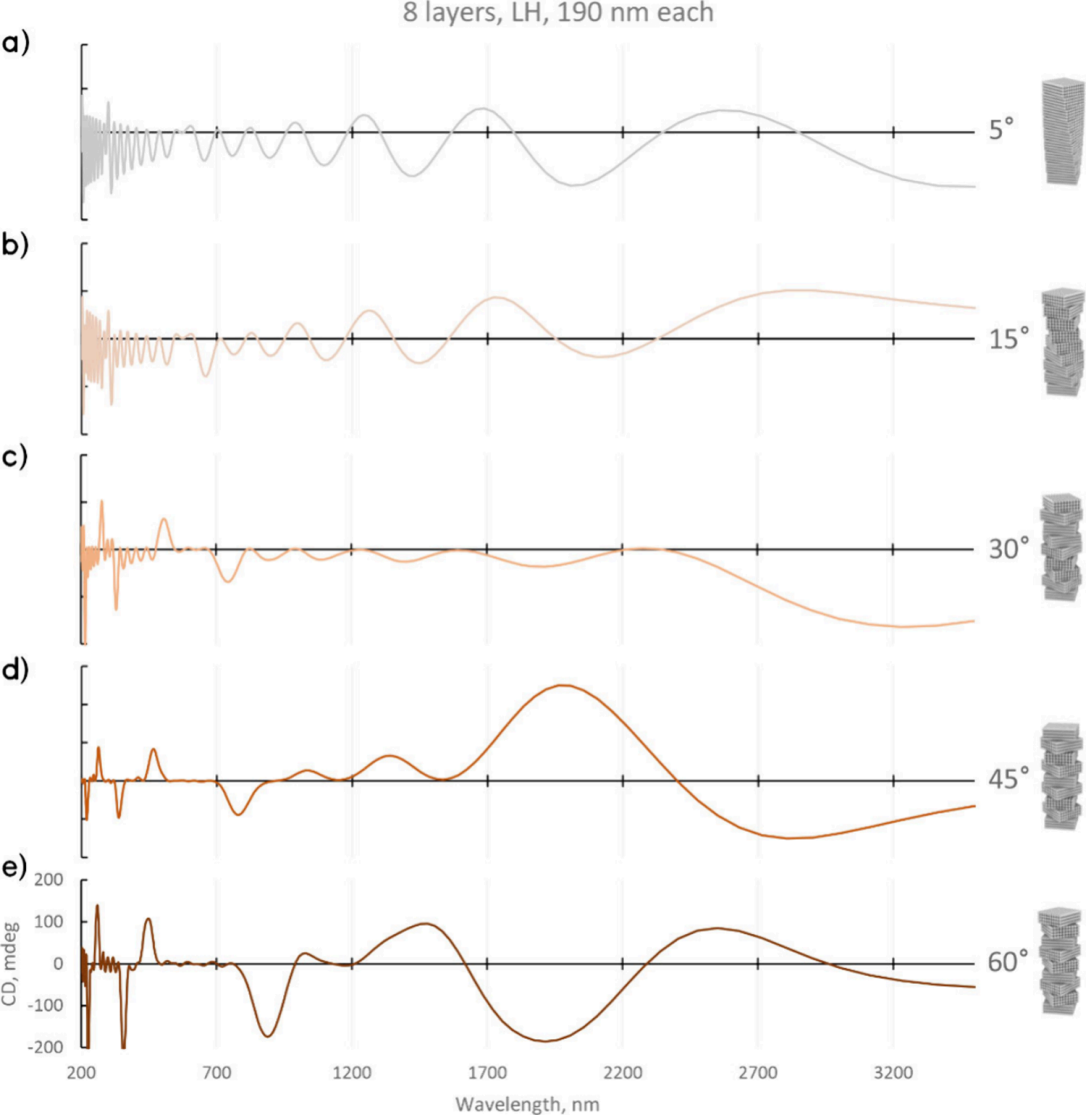
Simulated CD spectra for films with a single-layer thickness of 190 nm and different twisting angles with insets showing the corresponding twisted organization. (a, b) For smaller twisting angles at lower wavelengths, the signal is predominantly negative (RH). (c) With increasing angle step, positive (LH) peaks emerge. At (d) 45° and (e) 60°, the CD spectra contain both positive LH and negative RH peaks throughout the measured range.

First, simulations with constant layer thickness and increasing twisting angles were performed for the LH films with angles of 5°, 15°, 30°, 45°, and 60° (Figure 5). The simulated films with 5° and 15° twisting angles have predominantly RH CD at shorter wavelengths with strong interference fringes and a lack of distinct peaks. With increasing angles, both pronounced RH and LH peaks begin to emerge. At 30° (Figure 5c), LH peaks appear, and a broader RH signal shows in the NIR region (1700–3000 nm). At 45° and 60° twisting angles (Figure 5d,e), the spectra exhibit notably both LH and RH peaks throughout the observed ranges (see more discussion in the Supporting Information). From small to larger angles, the spectrum has transitioned from RH interference fringes to mixed CD signals with RH and LH peaks, which marks a profound change in the optical properties.

**Figure 6:**
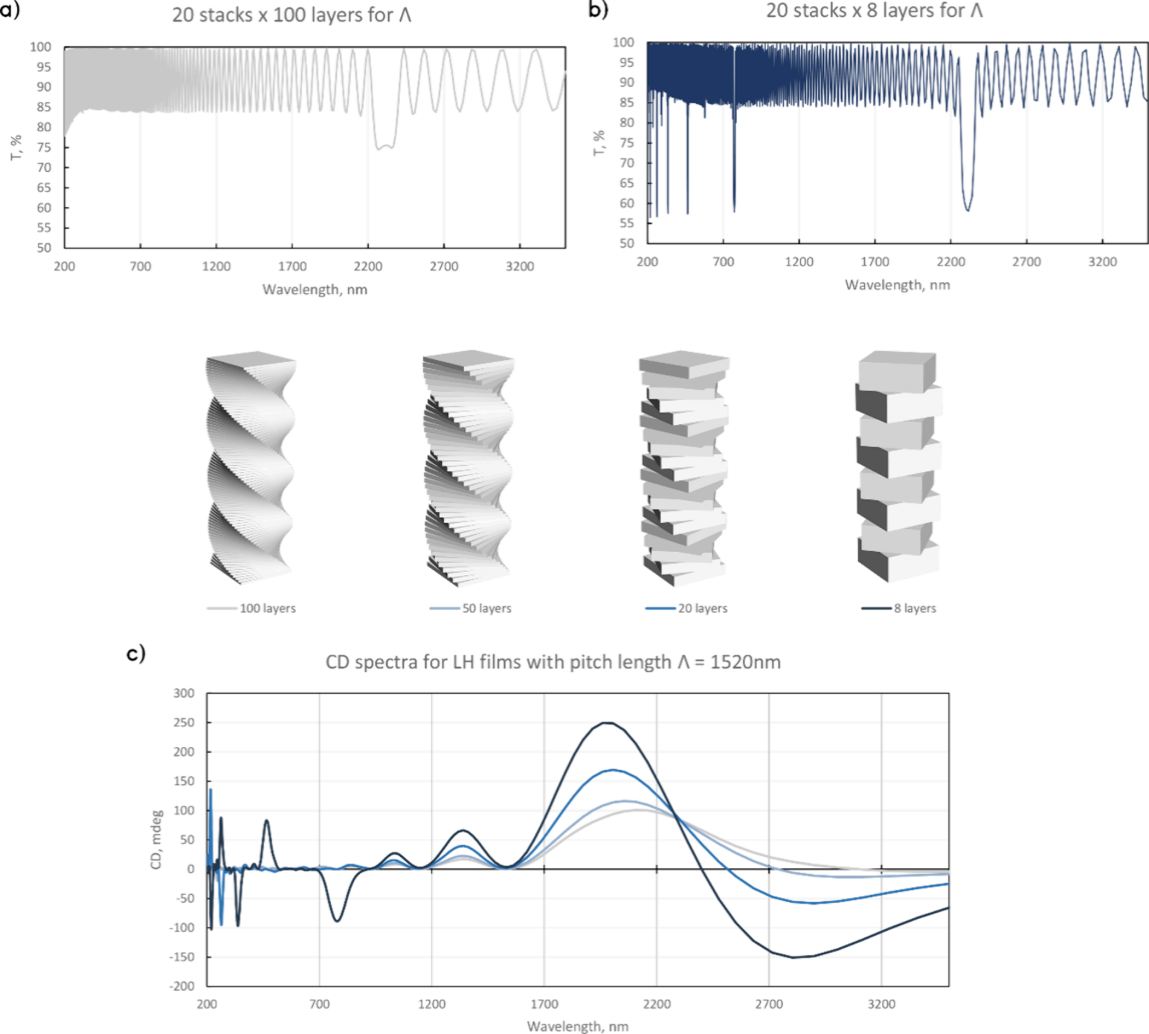
Simulated transmittance and CD for helical LH films with the same pitch length Λ = 1520 nm. (a) Transmittance for a 20 stack film (each stack length Λ has 100 layers). (b) Transmittance for a 20 stack film (each stack length Λ has 8 layers). (c) CD for 100, 50, 20, and 8 layer films in a single stack with length Λ.

Additionally, the emergence of multiple CD peaks was investigated via simulations with gradually increasing thickness of the single-layer slab from 1/100, 1/50, and 1/20 to 1/8 of the constant pitch length Λ (Figure 6). The 8 layer film (corresponding to 45° twisting angle) shows multiple LH and RH peaks with a main peak close to the expected condition for chiral nematic media:
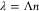
2where λ is the peak wavelength, Λ is the pitch length, and *n* is the average refractive index.^69,29^ However, the secondary peaks do not immediately follow the rules for harmonic interference peaks in lamellar dielectric mirrors^70,71^ or the condition for chiral nematic peaks (eq 2).

With an increasing number of layers, the single-handedness of the CD spectra becomes more strongly pronounced. At 20 layers, both LH and RH peaks are present at low wavelengths, and at 50 layers, the peaks have diminished in amplitude, while the main NIR peak remains. At 100 layers per pitch length, the RH peaks completely disappear, and the spectrum is dominated by a strong LH peak at the expected wavelength. A fundamental change occurs as the film becomes “more discrete” that triggers the spectral transition from a single bandgap to multiple photonic bandgaps with mixed handedness.

**Figure 7:**
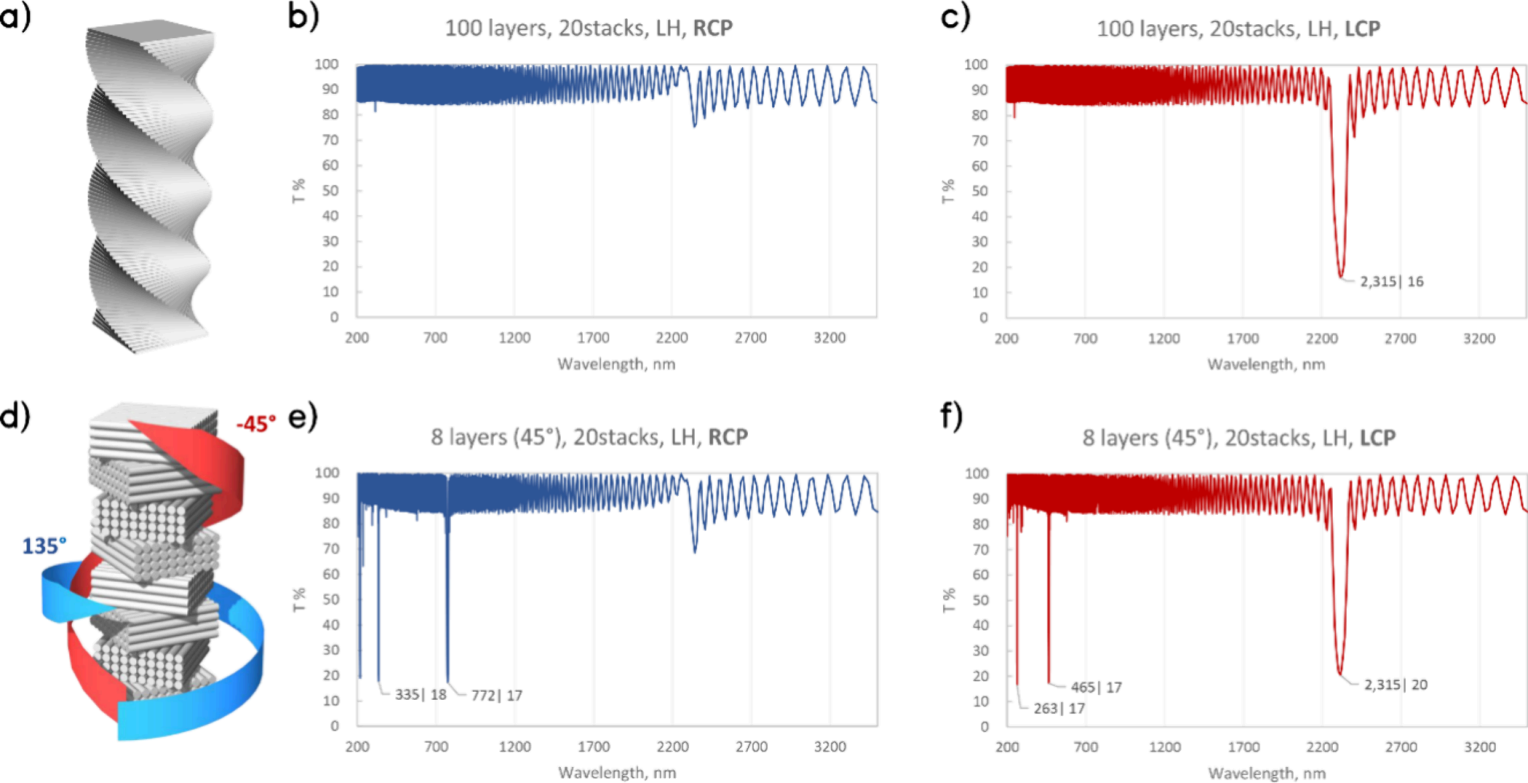
CPL simulations for a twisted film with 20 stacked pitch lengths. (a) Approximation of an LH continuous film with a 100 layer film with a layer thickness much smaller than the probed wavelengths and a twisting angle of 3.6°; transmittances of (b) RH CPL and (c) LH CPL. (d) Schematic of an LH 8 layer film with a 45° twisting angle and a layer thickness of Λ/8. The blue and red helical arrows indicate the presence of twisting in opposite directions: for in-plane mirror symmetry of a layer, a −45° twisting angle is equivalent to a 135° twisting angle. Transmittances of (e) RH CPL and (f) LH CPL for the 8 layer film.

Next, to decouple the optical response, simulations with LH and RH CPL transmittances were performed (Figure 7). For a continuous chiral nematic film, the LH CPL simulated transmittance shows a peak at the expected wavelength, while no activity was observed in RH CPL (Figure 7 a–c). Conversely, the twisted helical film formed by individual layer slabs in LH and RH CPL spectra shows peaks at distinctly different wavelengths (Figure 7 d–f), which cannot be attributed to Bragg reflection conditions, as pointed out above. A similar pattern has recently been observed in the work by Hu et al.^44^

### Circular Dichroism Appearance in Twisted Films

**Figure 8:**
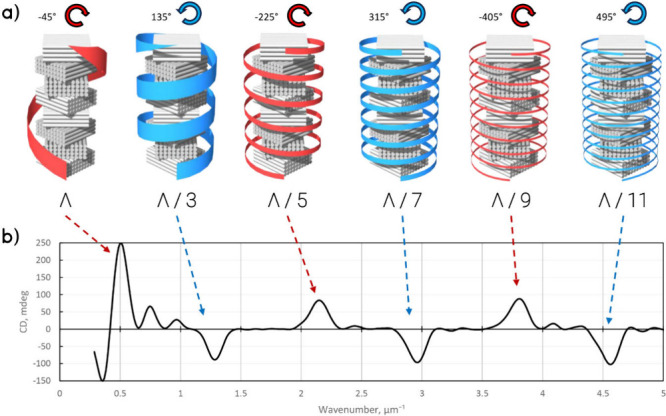
(a) Rotational harmonics and corresponding LH and RH CD peaks for the same twisted helical film. Helical arrows represent the helicity direction, red for LH and blue for RH. The pitch length that corresponds to the rotation step is indicated below each schematic. (b) CD spectrum plotted against the wavenumber with the peaks occurring at regular intervals.

Following the idea of helical duality further, the secondary CD peaks in the twisted CNC films occur at distinct twisting angles corresponding to “hidden” harmonic pitch lengths. Using the reflectance condition (eq 2) for chiral nematic media with infinitesimally small steps, we can estimate the pitch length with which individual peaks correspond. Each one of the identified pitch lengths can be attributed to a specific twisting angle with an initial phase shift. For example, a 465 nm LH peak corresponds to a 305 nm pitch length, which is 1/5 of the 45° pitch length Λ = 1520 nm, and can be formed by 225° twisting angles. This procedure can be extended for all peaks. Results for the 45° CCW twisting angle in LH films are provided in Figure 8.

After identifying the twisting angles for all observed bandgaps, it can be concluded that the peaks occur periodically with some base twisting angle α plus 180° due to the mirror symmetry in each single anisotropic layer. Thus, a condition for higher rotational harmonics will emerge for LH and RH peaks as
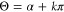
3where *k* is any integer. It is worth noting that we investigated two cases, 45° and 60°, which have different periodicities. For 45°, the bandgaps occur in Λ/3, Λ/5, Λ/7, Λ/9, Λ/11, and so on, while for 60°, they occur in −Λ/2, Λ/4, Λ/5, Λ/7, Λ/8, etc. It can be seen that the pattern sequence depends not only on the pitch length but also on the twisting step, and further investigations can be conducted for other twisting angles. The corresponding parameters derived from the simulated CD spectra are shown in Table S2.

In summary, the relationships discussed above (eqs 2 and 3) provide a straightforward explanation of where the CD peaks (bandgaps) will emerge for the case of twisted helical films and could be used to design the optical properties of the anisotropic materials in a programmed way. Furthermore, twisting control also provides the versatility to enhance or suppress any helicity and chirality and to achieve a fully 2D two-fold symmetric material.

**Figure 9:**
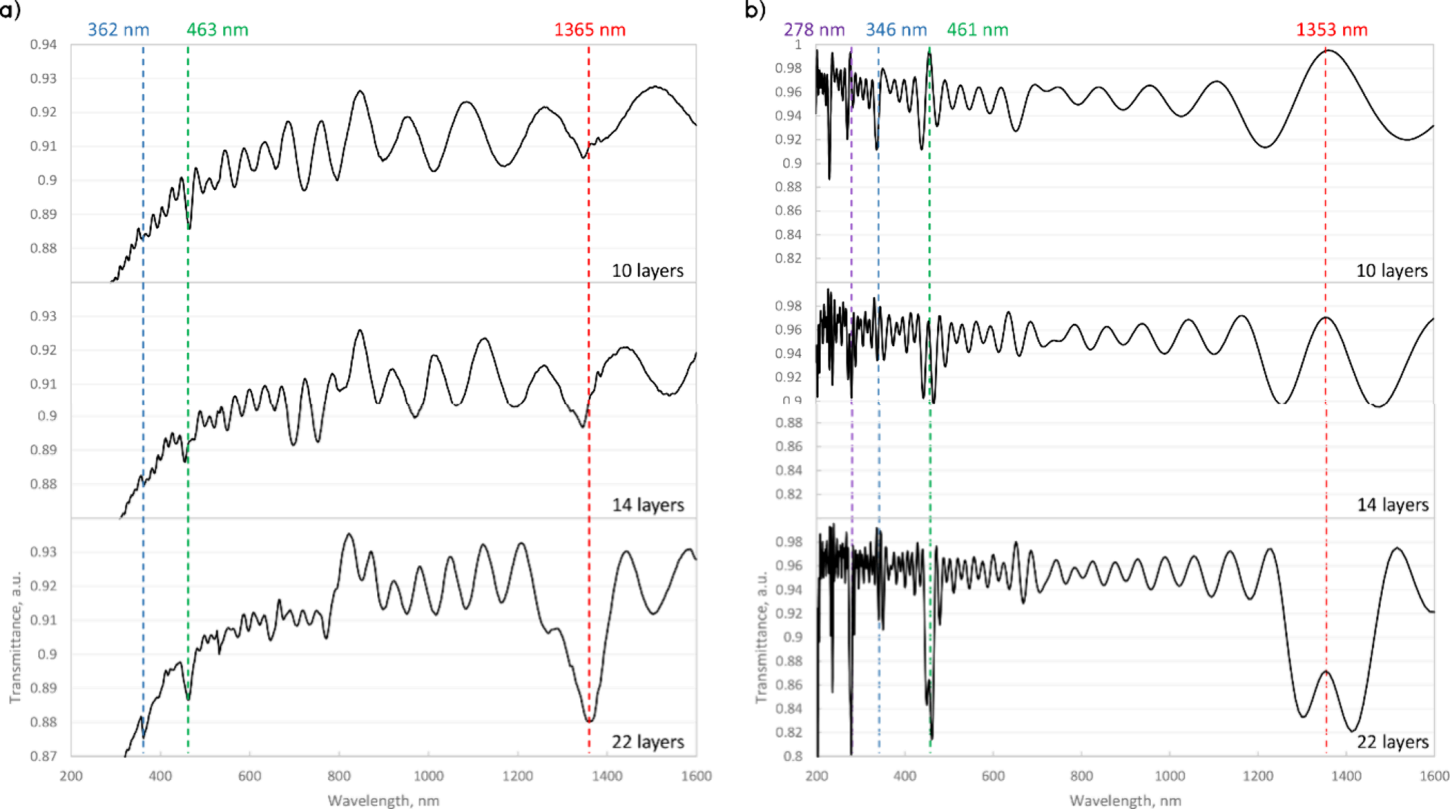
Transmittance spectra of the CNC anisotropic Bragg mirror CNC7.5-90 film with 10, 14, and 22 layers. The twisting angle between the adjacent layers is 90°. (a) Experimental results showing photonic peaks with increasing strengths emerging with the number of layers. (b) Simulated absorbance results for the Bragg mirror. In both (a) and (b), there are interference fringes. The dashed lines and text indicate the peak positions.

Finally, a film with perpendicularly oriented sequential anisotropic layers was produced and demonstrated with sample CNC7.5-90 (anisotropic Bragg stack). An absorbance peak at the expected wavelength in the NIR region corresponding to the Bragg condition emerges. Secondary higher harmonics occur at lower wavelengths under higher-order Bragg conditions (Figure 9a).

The FDTD simulations show peaks at approximately the same positions, with minor deviations below 15 nm (Figure 9b). Because the CNC material was modeled as a transparent dielectric throughout the entire measured spectrum, higher harmonic bandgaps are visible in the UV region (Figure 9b, violet, 278 nm).

In this case, the anisotropic Bragg mirror was designed to achieve a significant main bandgap in the NIR region (1363 nm), which has not been demonstrated with CNC materials to date (Figure 9). This film does not have any significant CD due to the presence of mirror symmetries (Figure S12). However, by controlling the layer thickness, other mid- and far-infrared wavelengths can also be targeted to result in highly specific optical filtration properties.

## Conclusion

This work introduced twisted photonic films with specific photonic bandgaps and circular dichroism that were printed via a facile top-down blade coating approach. The printed twisted helical CNC films show multiple circularly polarized bandgaps, subject to control by the layer thickness and twisting angle. The anisotropic Bragg mirror is at the other extreme, where the film loses chiroptical properties and behaves as a plain dielectric mirror, insensitive to circular polarization. These cases demonstrate the versatility of discrete layer twisting to engineer both circularly polarized phenomena and near-infrared reflection. With the help of simulations, it was demonstrated how the optics of the discrete helical twisted material differ from that of the self-assembled continuous chiral nematic film, resulting in circular dichroism spectra with multiple peaks of both left- and right-handedness.

We suggest that this work opens novel pathways for the preprogrammed stacking of anisotropic layers, a direction that has not been thoroughly explored, as it presents an opportunity for designing different layer sequences and periodicities. This is a potential alternative to lattice-based twisted metamaterials known for precision control of chiroptical properties.^72^ Shear-deposition of lyotropic suspensions applied to macroscale surfaces could help to avoid costly nanofabrication methods^73^ and with this brings twisted materials closer to large-scale applications in response to the high demand for materials with an advanced circularly polarized response in biological and chemical sensors, filters, emitting devices, and polarizers.

Furthermore, complex stacking patterns of twisted 2D materials with high refractive indices (e.g., metal oxides, MXenes) could result in 3D materials with periodic and very high optical contrasts through the emergence of new symmetries,^74^ potentially yielding higher nonlinear chiroptical properties. Other possibilities include the introduction of responsive subphases for the interlayers, 3D photonic structuring via nanopatterning with customized deposition tools, the inclusion of plasmonic nanoparticles, and functional interlayer materials with high optical contrast for architected coatings with potential for tailored energy conservation, light protection, optical communication, and enhanced visibility in a wide spectral range including mid- and far-infrared.^75^

## Supporting Information Available

Additional experimental characterization methods, results, and procedure descriptions (PDF)
