# Supplementary material for: Printed Twisted Thin Films with Near-Infrared Bandgaps and Tailored Chiroptical Properties
Source: ACS Appl Opt Mater. 2024 Nov 21;2(12):2540–50. doi: 10.1021/acsaom.4c00386 (PMC11686506; doi:10.1021/acsaom.4c00386)
Supplement: Supplementary file 1 — ot4c00386_si_001.pdf [file ot4c00386_si_001.pdf]

Supporting Information

Printed Twisted Thin Films with Near-Infrared Bandgaps and Tailored Chiroptical Properties

Botyo Dimitrov[1], Daria Bukharina[1], Valeriia Poliukhova[1], Dhriti Nepal[2], Michael E. McConney[2], Timothy J. Bunning[2], Vladimir V. Tsukruk[1*]

[1] School of Materials Science and Engineering, Georgia Institute of Technology, Atlanta, Georgia 30332-0245, United States

[2] Air Force Research Laboratory, Wright-Patterson Air Force Base, Dayton, Ohio 45433, United States

[*] vladimir@mse.gatech.edu

Cellulose Nanocrystals (CNC)

To measure the CNC size, a dilute suspension was spin-coated on a silicon wafer and scanned on AFM with tips with a 2 nm radius. Approximately 100 CNC particles were measured. The results are provided in Table S1. These values are in good agreement with previous reports in literature.[1]

Table S1. Average Dimensions of Cellulose Nanocrystals

Diameter (nm)	Length (nm)	Aspect Ratio
4.1 ± 1	135 ± 52	33

Blade Coating Procedure

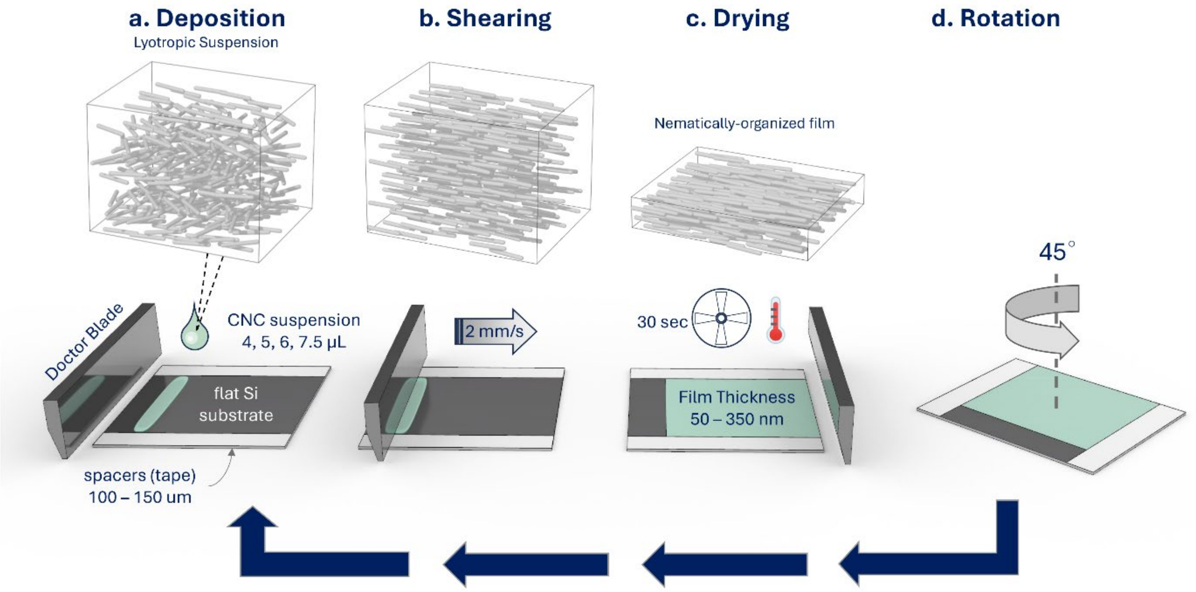

Figure S1. a) CNC suspension is deposited on an atomically flat Si wafer. We assume the presence of lyotropic phase.[2] b) The blade is applied and separated from the substrate surface by two or three layers of tape with a total thickness of 100-150 µm. The blade coater tool is then started to move the blade on top of the substrate surface at a constant rate to shear the CNC suspension. c) The coating process results in a wet nematic layer, which upon drying, evaporates the water solvent and collapses the CNC phase to a solid film with a thickness on the order of 100 nm. d) Finally, the substrate can be rotated, and the procedure – repeated, to achieve a multilayer twisted film.

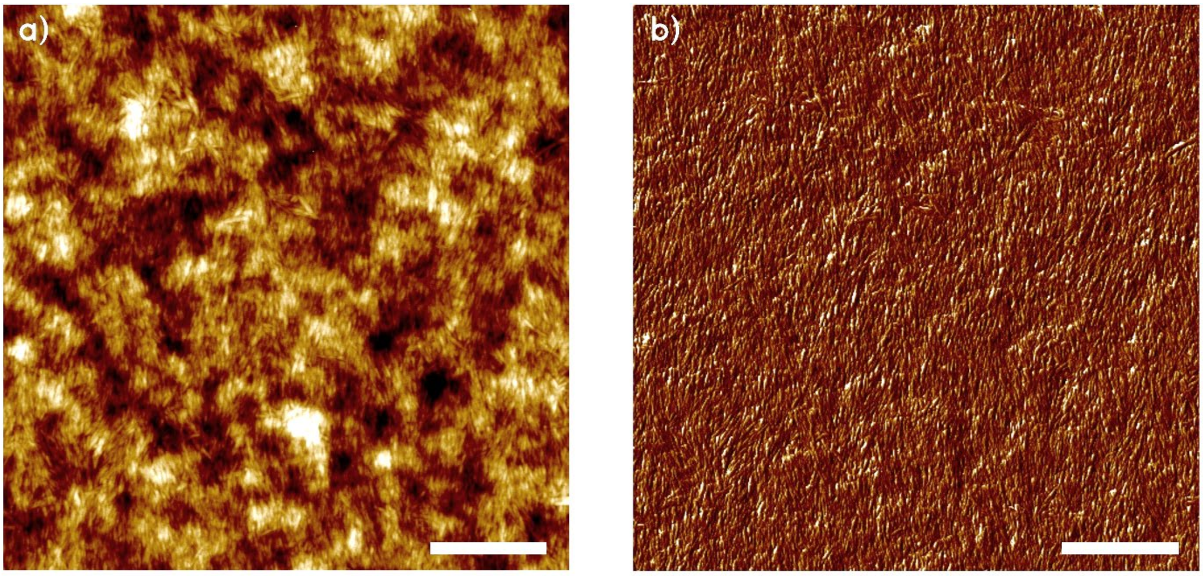

Figure S2. AFM topographical (a) and phase (b) images of individual layer. Scale bars are 1 µm.

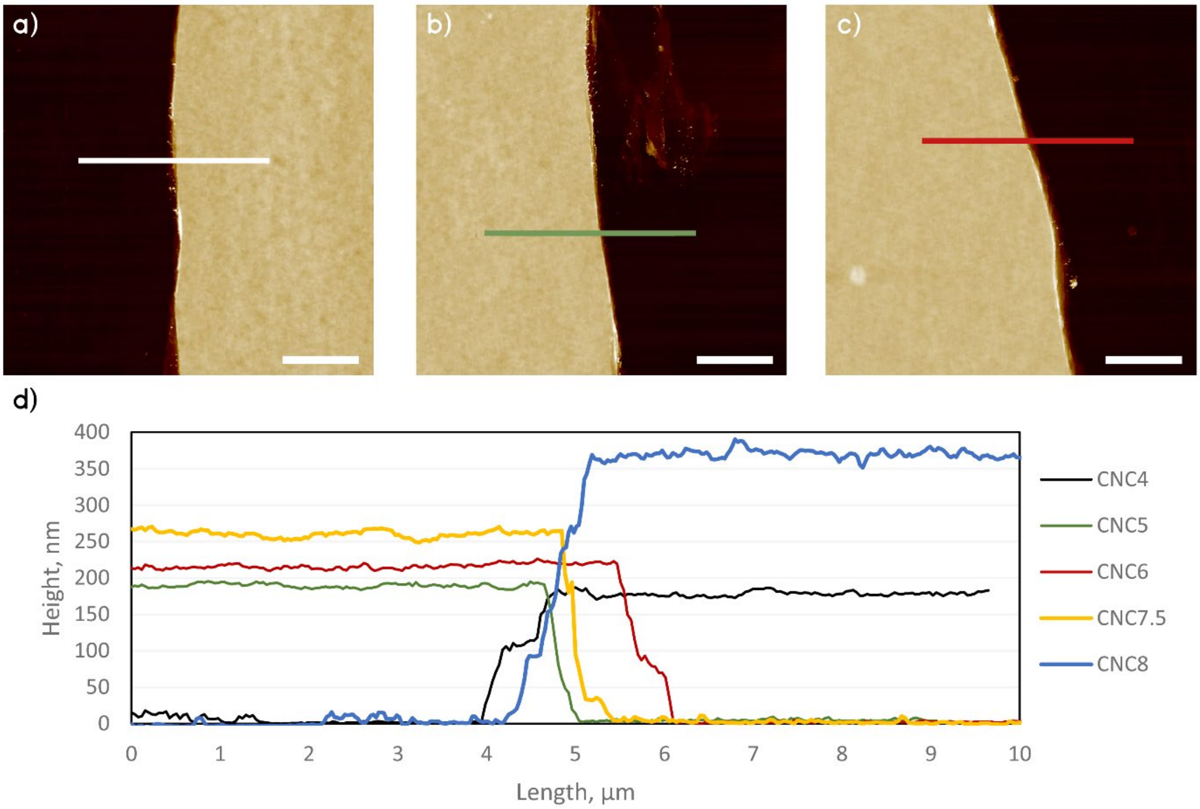

Figure S3. AFM images of the CNC4 (a), CNC5 (b), and CNC6 (c) single bilayer films after razor-blade scratch. Scale bars are 4 µm. d) Cross-sectional height data – black, green, red, yellow, and blue correspond to CNC4, CNC5, CNC6, CNC7.5, and CNC8, respectively.

Ellipsometry measurements

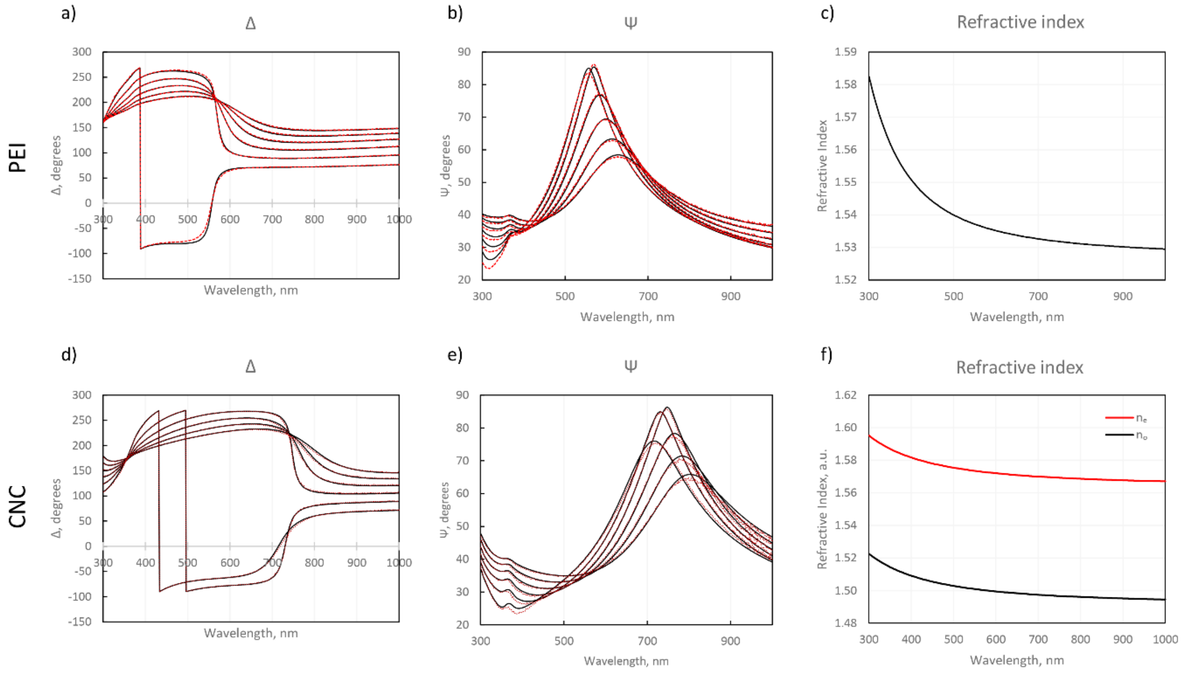

Figure S4. Ellipsometry measurements and modeling as a Cauchy dielectric. a, b, c) Delta, Psi fits, and refractive index as a Cauchy material for a PEI film on a Si wafer substrate. d, e, f) Delta, Psi fits, and n values for single shear-deposited CNC layer on Si substrate modelled as anisotropic Cauchy material.

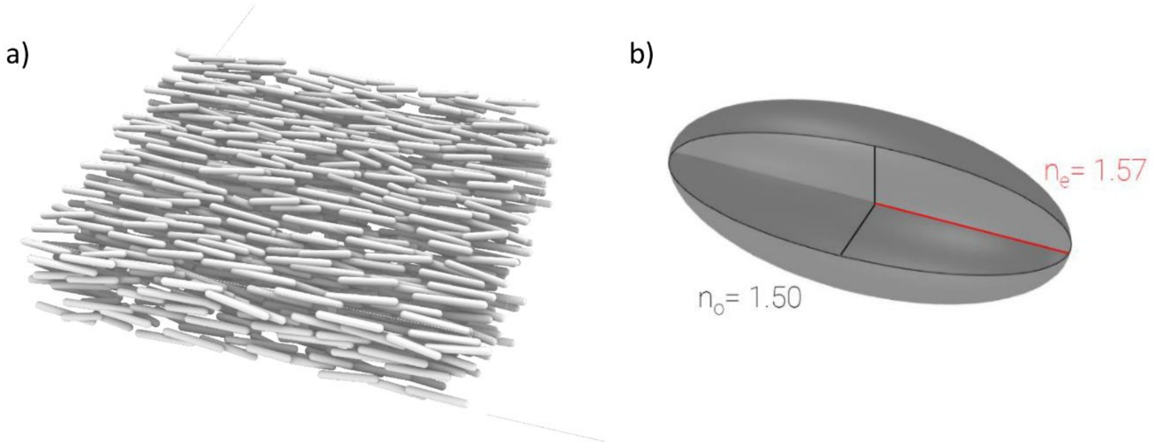

Figure S5. a) 3D geometric representation of the single nematically-organized layer. b) Refractive index ellipsoid (not to scale) for 700 nm wavelength with ordinary and extraordinary refractive indices color-coded in black and red respectively.

Orientation Order Parameter

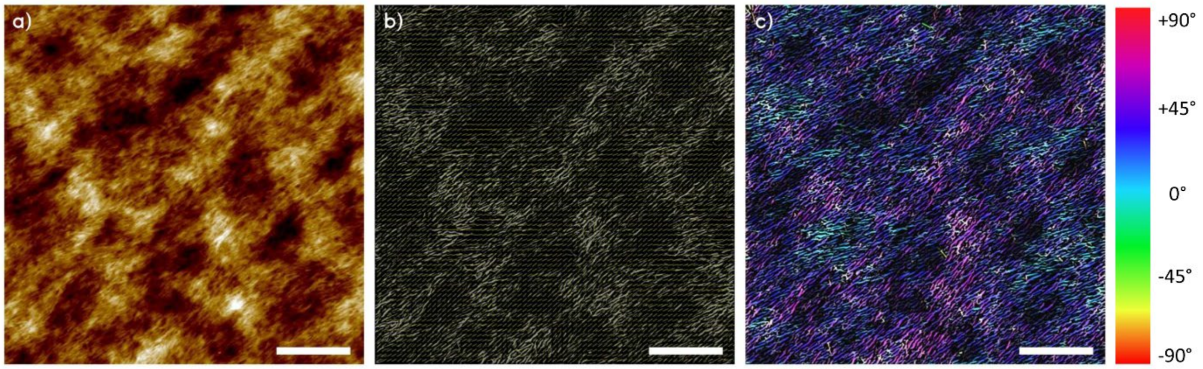

Figure S6. AFM images and CNC orientation analysis. CNC7.5 film at 45deg blade-coating direction. a) AFM image of blade-coated CNC films on Si substrate. b) Vector field superposition using the Orientation plug-in for FiJi-ImageJ. c) Orientation analysis coloration using the same software. Orientation legend on the right.

Orientational Order Parameter was calculated for the single-layer CNC4, CNC5, CNC6 films, and two-layer CNC7.5 (Fig. S6) films. We are interested in investigating the in-plane anisotropy of the films. Thus, the 2D Order Parameter (planar order parameter, Chebyshev order parameter)[3] is more pertinent compared to 3D Hermann Orientational Order Parameter, because of the direct evaluation of in-plane alignment.[4] The 2D Order Parameter is defined as $S_{2D} = 2\langle cos^2\,\theta\rangle - 1$, where θ is the angle between the nanocrystal long axis and the defined director vector, and was calculated by processing AFM surface height images using the following procedure. First, the image (Fig. S6a) is imported into the FiJi-ImageJ software and converted to 8-bit file format. The Tubeness operation is applied to accentuate localized height differences resulting in a sharper image. Next, the Vector Field command available in the Orientation plug-in[5] is applied to result in a superimposed vector depiction (Fig. S6b). A CSV file with the orientational angle for every position in the vector field is automatically generated and used for the order parameter calculation. Finally, the Orientation Analysis from the same plug-in is used to colorize the image for visual confirmation of the results (Fig. S6c).

Roughness Measurements

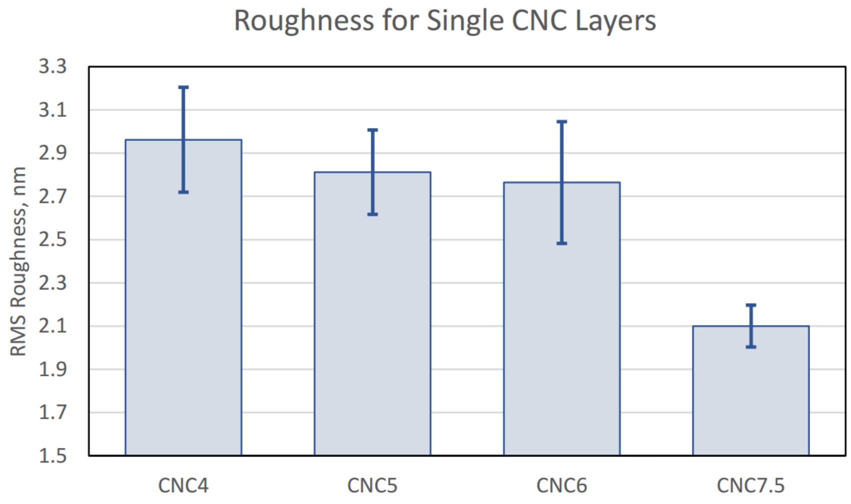

Figure S7. RMS Roughness as measured from AFM Images in 1x1 µm regions on single-bilayer PEI-CNC films.

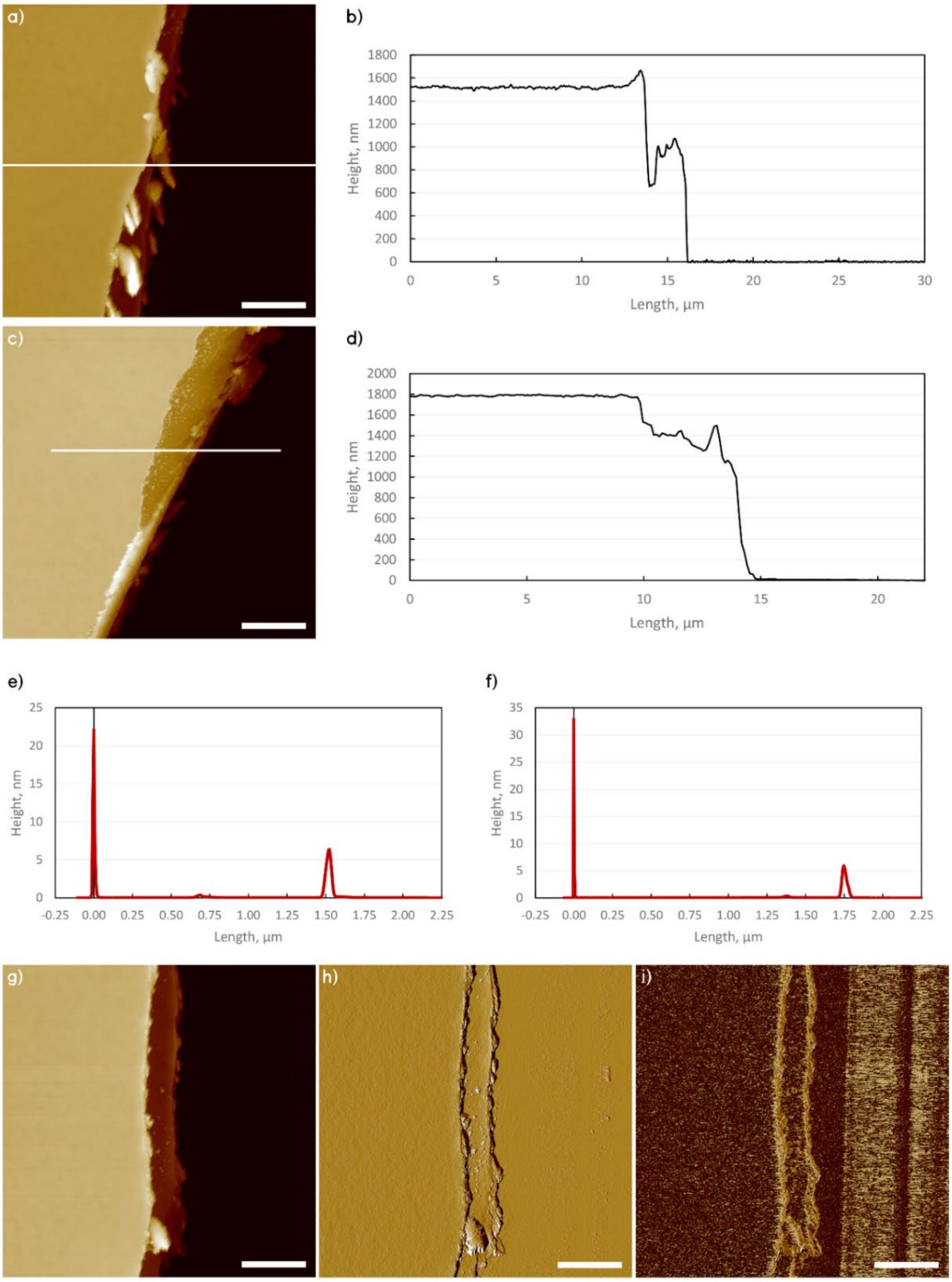

Figure S8. AFM image of scratch-cut CNC4-45 film (a) and CNC7.5-45 film (c), and the respective cross-section height data (b, d). The scale bars are 6 µm long. Both images show the substrate surface (darker) and the CNC film surface (lighter) with the presence of fragments occurring in the process of razor-blade cutting. These fragments are weakly attached to the bulk film surface leading to a fuzzy appearance in the AFM height sensor image. Depth histograms of CNC4-45 (e) and CNC7.5-45 (f) films. g,h,i) Scratch-test of a CNC film with height sensor, amplitude error, and phase images. The amplitude error (h) shows the largest deviations corresponding to the fuzzy portions of the height sensor data (g)

along the edge cut. The phase image (i) indicates the presence of a different phase corresponding to the substrate surface.

Light Interference Fringes for Printed Films

UV-Vis-NIR spectroscopy of the twisted helical films shows nearly complete transparency in the visible and NIR ranges, with modestly decreased transmittance in the UV range (Fig. S9). On average, the transmittance is 91% throughout the spectra, with the exception of the UV region, where it sharply decreases.

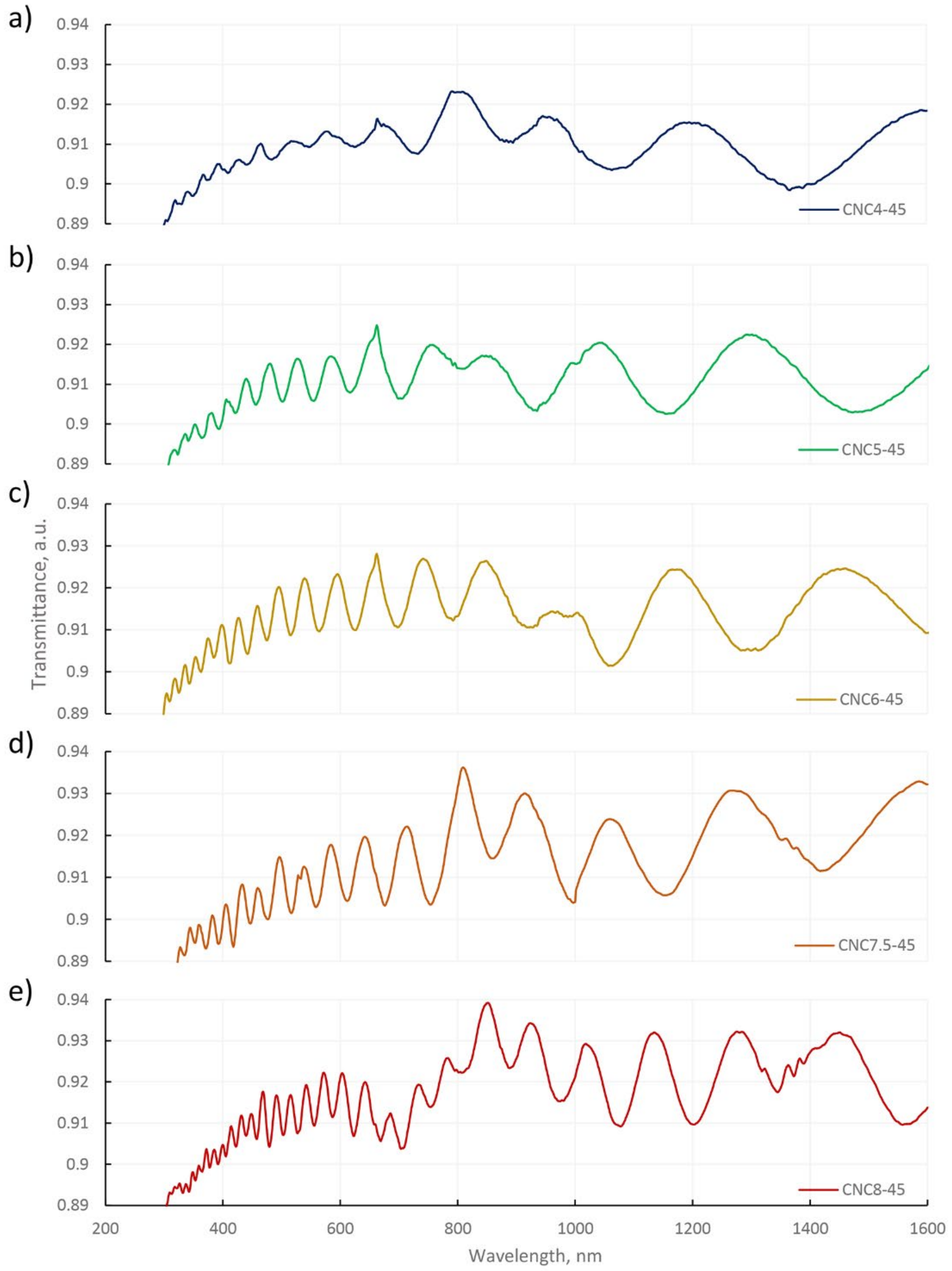

Figure S9. UV-Vis-NIR spectra of LH twisted helical films with increasing layer thickness for CNC4-45 (a), CNC5-45 (b), CNC6-45 (c), CNC7.5-45 (d), and CNC8-45 (e) films. Interference fringes are observed throughout the entire measured range with a decreasing period corresponding to the increasing thickness of the films.

The presence of interference fringes indicates parallel upper and lower surfaces of the film and roughness is negligible for the probed wavelengths. The low surface roughness was confirmed by AFM measurements indicating an RMS roughness of 2-3 nm within 1x1 µm surface areas (Fig. S7). These values are very small compared to overall film thickness (around 1%), which is around 100-250 nm. In fact, all films are highly transparent with transmittance across the whole spectral range within 90-94%.

In a complementary approach, the film thickness can be measured using Swanepoel's method[6] combined with FFT analysis (Fig. S10, S11) using the already obtained refractive index from ellipsometry data (Fig. S4). This method, independent of the extinction coefficient of the material, should be valid for multilayer media considering the dominant contribution for interference at the interfaces between air and CNC film and between CNC film and fused silica substrate[7], rather than the CNC interlayers. The thickness of the multilayer film was measured for CNC4-45 and CNC7.5-45 showing 1560 and 2160 nm average values that are comparable to AFM measurements (1550 and 1750 nm respectively) with the deviation in the thicker film for higher concentration suspensions can be attributed to batch differences.

Film thickness using Swanepoel's method and Fast Fourier Transform

Swanepoel's method for obtaining the film thickness is given by:

$$d = \frac{\lambda_1 \lambda_2}{2(\lambda_1 n_2 - \lambda_2 n_1)} \qquad \text{(Eq. S1)}$$

where d is the total film thickness, λ_1 and λ_2 are the peak positions and n_1 and n_2 are the respective refractive indices. Refractive index data was taken from ellipsometric measurements.

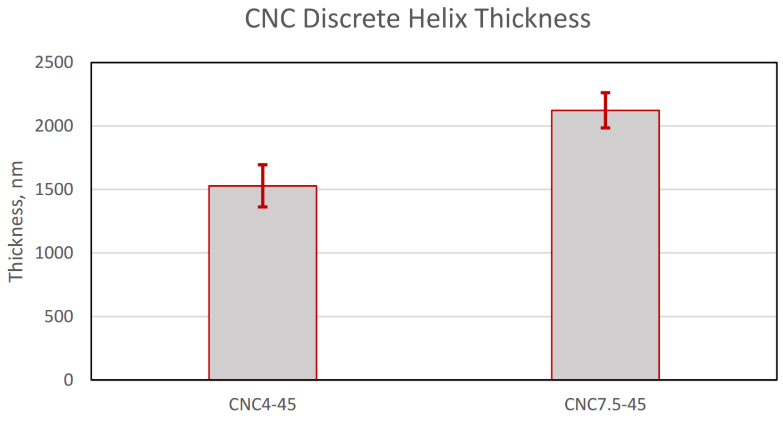

Figure S10. Thickness distribution as measured from UV-Vis-NIR spectra peak distance and calculated using the refractive index values established from the spectroscopic data. The distance between couples of adjacent peaks in the wavelength range 300-1000 nm was taken into account. The results for CNC7.5-45 show a larger thickness compared to AFM measurements due to inconsistencies between CNC suspension batches.

Fast Fourier Transform (FFT) was used to determine the optical thickness using the procedure described in Quinten.[8] The transmittance data against the wavenumber was loaded in the *Origin 2022 Software*, and the built-in FFT function was applied. The resulting peak of the power spectrum was calculated by:

$$d = \frac{m}{2n\,(\upsilon_{max} - \upsilon_{min})} \qquad \text{(Eq. S2)}$$

where m is the Fourier index, n is the refractive index, v_{min} and v_{max} are the lowest and highest measured wavenumbers, respectively, and d is the thickness. For a constant refractive index of 1.54, the results are 1.56 µm for the CNC4-45 and 2.16 µm for the CNC7.5-45 films, consistent with Swanepoel calculations.

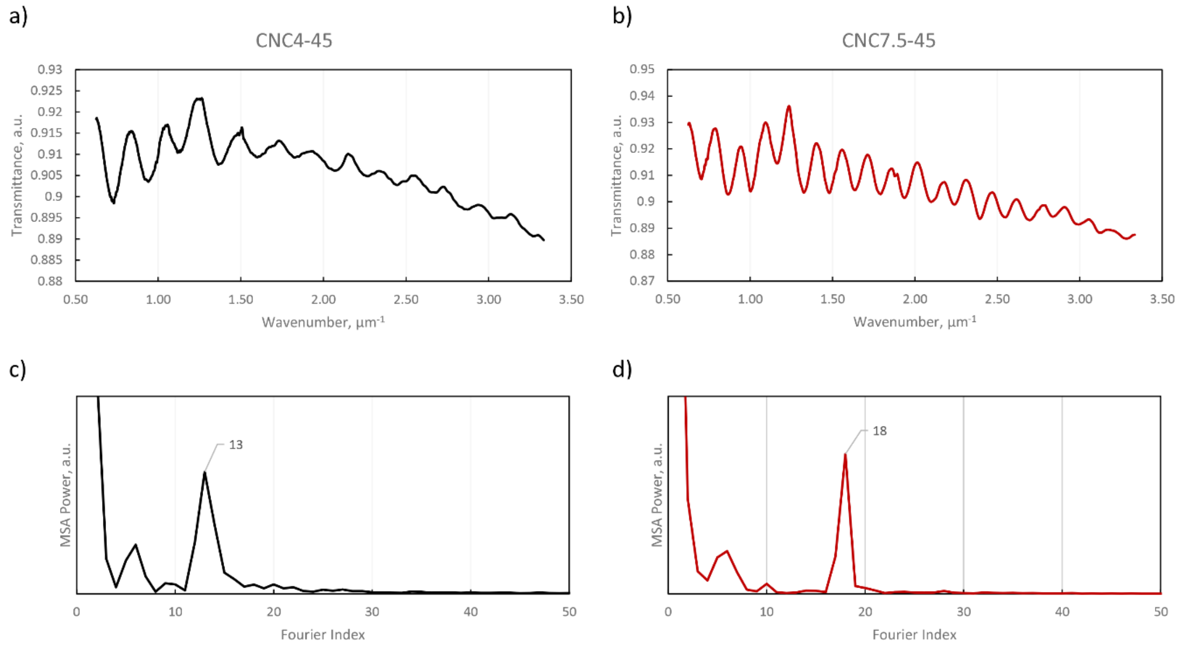

Figure S11. Interference fringes (a,b) and Fourier Power spectra (c,d) for CNC4-45 and CNC7.5-45 films.

Emergence of bandgaps at low twisting angles

Handedness due to rotational harmonics can also explain why the simulated CD spectra in Fig. 5 (main text) for 5° (a) and 15° (b) steps show RH behavior at low wavelengths. For both cases, we would expect the rule (Eq. 3, main text) to also be true and dominated by a single LH peak at the respective pitch length. However, the RH peaks will correspond to small pitch lengths, due to the proximity to a 180° twisting step and therefore will appear at shorter wavelengths. Therefore, in the LH films with small step, we will see negative CD and vice versa. The same approach can be used to explain the “sudden” emergence of peaks in the simulated transition from 100 to 8 layers per pitch length (Fig. 6c, main text). Mixed sign handedness of CD signals will be present for all cases of printed twisted films composed of anisotropic discrete blocks. However, at extreme limits with a large number of layers (100

and 50) the RH peaks will occur in the UV range due to large RH twisting angles (small LH angles) and corresponding small pitch lengths.

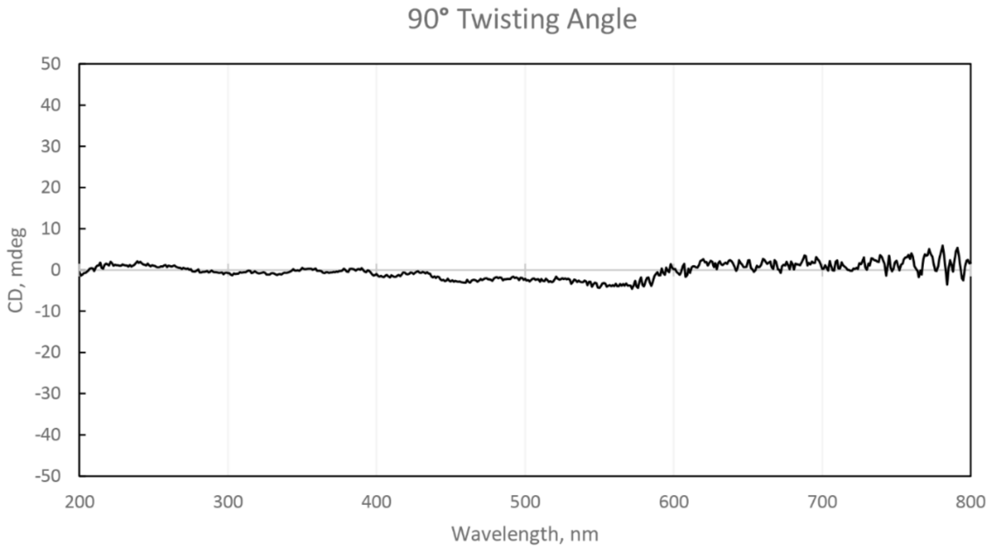

Figure S12. CD spectrum of anisotropic Bragg stack 8-layer CNC7.5-90 film. The film shows negligible CD activity.

Table S2. CPL transmittance peaks and the respective calculation for a single layer thickness of 190 nm for twisting angles -45°/135° and -60°/120° based on simulated data. The peak wavelength is linearly proportional to the pitch length. The so-calculated pitch length can be used to further estimate the number of layers needed to form a discrete helix with respective twisting angle **θ**. Since rotation is periodic and each layer has 2 planar mirror symmetries, any angle **θ** can be expressed as a base angle **α** added to ***kπ***, which explains the corresponding peak positions.

-45°, 135° rotation

Source Polarization	Peak (nm)	n	Λ (nm)	Base layer (nm)	# layers	Θ (°)	Periodicity per 8 layers
LCP	2315	1.523	1520	190	8	-45	1
LCP	465	..	305	..	1.6	-225 = (-45-**180**)	5
LCP	263	..	173	..	0.91	-395 = (-45-**350**)	9
RCP	772	..	507	..	2.66	135	3
RCP	335	..	220	..	1.16	315 = (135+**180**)	7
RCP	218	..	143	..	0.75	480 = (135+**345**)	11

-60°, 120° rotation

Source Polarization	Peak (nm)	n	Λ (nm)	Base layer (nm)	# layers	Θ (°)	Periodicity per 6 layers
LCP	2315	1.523	1520	190	6	-60	1
LCP	580	..	381	..	1.5	-240 = (-60-**180**)	4
LCP	335	..	220	..	0.87	-420 = (-60-**360**)	7
RCP	1155	..	758	..	3	120	2
RCP	465	..	305	..	1.2	300 = (120+**180**)	5
RCP	294	..	193	..	0.76	480 = (120+**360**)	8

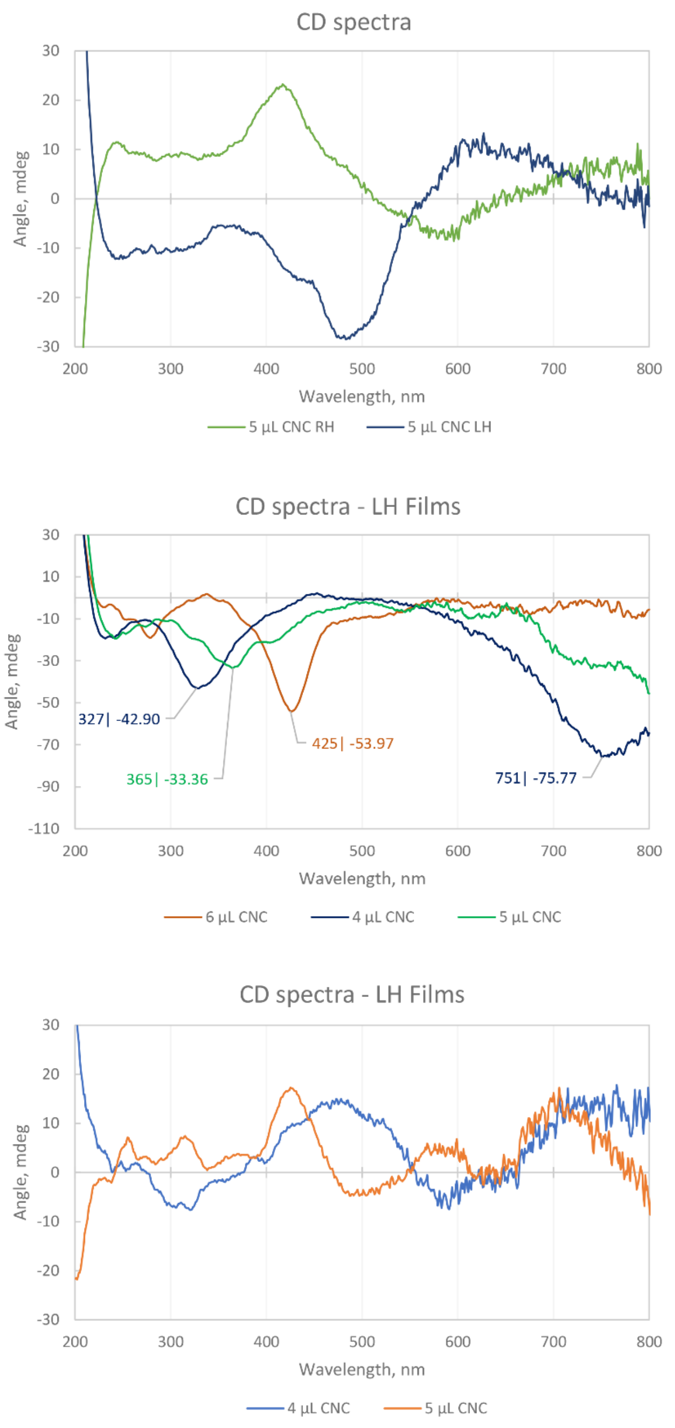

Figure S13. CD measurements of films assembled from different batches. Top – LH and RH twisted films. Middle – twisted films with increasing thickness (4, 5, 6 μL). Bottom – twisted films with 4 and 5 μL.

Further studies were done with other CNC batches with the same 45° twist angle (Fig. S14). LH and RH films show nearly symmetrical CD (Fig. S14 top). Films with increasing thickness also show red-shift with increasing thicknesses, although the positive (LH) peaks are missing (middle). The occurrence in the negative (RH) peaks approximately follows the expected positions. However, the

lack of LH peaks can be attributed to the lack of order for the respective pitch lengths. Finally, “bad” batches (with failed CNC hydrolysis), lack regularity in the CD response due to a lack of alignment within layers (bottom).

References

[1] Bushell, M.; Meija, J.; Chen, M.; Batchelor, W.; Browne, C.; Cho, J.-Y.; Clifford, C. A.; Al-Rekabi, Z.; Vanderfleet, O. M.; Cranston, E. D.; Lawn, M.; Coleman, V. A.; Nyström, G.; Arcari, M.; Mezzenga, R.; Park, B. C.; Shin, C.; Ren, L.; Bu, T.; Saito, T.; Kaku, Y.; Wagner, R.; Johnston, L. J. Particle size distributions for cellulose nanocrystals measured by atomic force microscopy: an interlaboratory comparison. *Cellulose* **2021**, *28* (3), 1387–1403. DOI: 10.1007/s10570-020-03618-4.

[2] Kádár, R.; Spirk, S.; Nypelö, T. Cellulose nanocrystal Liquid Crystal Phases: progress and challenges in characterization using rheology coupled to optics, scattering, and spectroscopy. *ACS Nano* **2021**, *15* (5), 7931–7945. DOI: 10.1021/acsnano.0c09829.

[3] Kaniyoor, A.; Gspann, T. S.; Mizen, J. E.; Elliott, J. A. Quantifying alignment in carbon nanotube yarns and similar two-dimensional anisotropic systems. *J. Appl. Polym. Sci.* **2021**, *138* (37). DOI: 10.1002/app.50939.

[4] Bukharina, D.; Kim, M.; Han, M. J.; Tsukruk, V. V. Cellulose Nanocrystals’ Assembly under Ionic Strength Variation: From High Orientation Ordering to a Random Orientation. *Langmuir* **2022**, *38* (20), 6363–6375. DOI: 10.1021/acs.langmuir.2c00293.

[5] Rezakhaniha, R.; Agianniotis, A.; Schrauwen, J. T. C.; Griffa, A.; Sage, D.; Bouten, C. V. C.; Van De Vosse, F. N.; Unser, M.; Stergiopulos, N. Experimental investigation of collagen waviness and orientation in the arterial adventitia using confocal laser scanning microscopy. *Biomech. Model. Mechanobiol.* **2011**, *11* (3–4), 461–473. DOI: 10.1007/s10237-011-0325-z.

[6] Swanepoel, R. Determination of the thickness and optical constants of amorphous silicon. *J. Phys. E: Sci. Instrum.* **1983**, *16* (12), 1214–1222. DOI: 10.1088/0022-3735/16/12/023.

[7] Cranston, E. D.; Gray, D. G. Morphological and optical characterization of polyelectrolyte multilayers incorporating nanocrystalline cellulose. *Biomacromolecules* **2006**, *7* (9), 2522–2530. DOI: 10.1021/bm0602886.

[8] Quinten, M. A Practical Guide to Optical Metrology for Thin Films; John Wiley & Sons, Incorporated, 2012. DOI: 10.1002/9783527664344.
